# From pain to policy: Improving endometriosis awareness, diagnosis, and treatment

**DOI:** 10.1371/journal.pmed.1004981

**Published:** 2026-03-02

**Authors:** Alexandra Tosun, on behalf of the PLOS Medicine Staff Editors

**Affiliations:** Public Library of Science, Berlin, Germany

## Abstract

In recognition of Endometriosis Awareness Month, this Editorial by Alexandra Tosun discusses why raising awareness and education of endometriosis, as well as increasing funding and research for this systemic condition, are imperative to redefining pain, equity, and investment in women’s health.

## Teaser

Despite affecting 190 million women worldwide, endometriosis remains underdiagnosed, under-researched, and underfunded. Tackling awareness gaps, diagnostic delays, and inadequate treatment requires earlier education, increased funding, and recognition of endometriosis as a systemic disease—redefining pain, equity, and investment in women’s health.

“Your pain is normal”, “all women experience this”, “you have a low pain tolerance”—statements painfully familiar to many patients on their journey to an endometriosis diagnosis. Despite affecting around 190 million women of reproductive age worldwide, endometriosis remains heavily underdiagnosed, as symptoms are too often normalized, dismissed, or mistaken for other conditions. To raise awareness, advance clinical understanding, and strengthen support for those affected, March marks Endometriosis Awareness Month.

Although the Endometriosis Association designated March as Endometriosis Awareness Month in 1993 to promote global recognition, it was not until the 2010s and through social media that endometriosis gained wider attention. Endometriosis—a chronic inflammatory condition where cells similar to those in the lining of the uterus grow in other parts of the body—is often accompanied by symptoms such as severe menstrual pain, heavy menstrual bleeding, chronic pelvic pain, infertility, abdominal bloating, and nausea [1]. Despite these often-debilitating symptoms, patients typically face excessive times to diagnosis of between 5 and 12 years [2], during which each menstrual cycle may aggravate disease progression and pain. The diagnostic delay is frequently compounded by lack of awareness, education, and social stigma on both the patient and healthcare side. With menstrual pain being commonly normalized, many patients downplay their symptoms or are afraid of doctors’ responses. Clinicians, on the other side, may struggle to initiate conversations around treatment options and their long-term consequences, stemming from limited evidence and limited coverage of the topic in medical training.

To date, no definitive cure for endometriosis is available and access to early diagnosis and treatment is limited in many settings [1]. In low- and middle-income countries, where high out‑of‑pocket costs, poor public awareness and stigma, shortages of specialist providers and health systems constraints converge, diagnosis and treatment become even more challenging. Oftentimes, disease management focuses on symptom control and limiting long-term complications, focusing on pain management with nonsteroidal anti-inflammatory drugs such as ibuprofen and/or hormonal medicines such as the contraceptive pill. Surgical treatment can remove endometriosis lesions, adhesions, and scar tissue, but when endometrial implants involve multiple organs, these become among the most complex and demanding procedures in gynecologic surgery.

However, barriers to improving endometriosis diagnosis and treatment arise even before patients present with symptoms; it begins with a profound lack of understanding due to insufficient research funding. Between 2019 and 2023, less than 1% of healthcare research and development funding went to female-specific conditions, highlighting severe underinvestment in women’s health generally [3]. The scarcity of scientific data often results in less interest by funders, perpetuating a cycle of underrepresentation and risk aversion. However, efforts to break this cycle are gaining momentum: Philanthropic donations, such as those from the Ainsworth family, who have together committed $50 million over 10 years to establish the Ainsworth Endometriosis Research Institute (AERI) at the University of New South Wales, aim to pioneer breakthroughs in diagnostics and the creation of precision-based treatments [4].

Such private commitment points to the urgency of the issue and the extent of systemic neglect where policies have failed to support awareness and research. To date, Australia and France are the only two countries with stand-alone national action plans (NAPs) for endometriosis [5]. While motions for endometriosis NAPs have been raised in other countries, such as Sweden, Canada, and Germany—and some view this policy momentum as promising—it remains undebatable that these steps alone are not enough. Recent studies highlight the extensive comorbidity burden associated with endometriosis. Endometriosis is associated with an almost 3-fold increased risk of irritable bowel syndrome [6], a 23% higher risk of developing cardiovascular disease [7], and women with endometriosis are more than twice as likely to suffer from depression, eating disorders, and anxiety [8]. A large US multi-center study identified hundreds of conditions associated with endometriosis, including genitourinary disorders, neoplasms, and autoimmune diseases, exposing the complexity and heterogeneity of the disease [9]. Therefore, rather than endometriosis being treated as an isolated gynecologic condition, it should be addressed as a systemic disease with long-term cardiovascular, metabolic, and mental health trajectories, with better integration into chronic disease frameworks. Such an approach, alongside increased funding for endometriosis research, could help redefine pain, equity, and investment in women’s health.

While policymakers fail to prioritize women’s health on their agenda, economists appear to have picked up on the potential of women’s health as a business model. A report by the Boston Consulting Group highlighted that “Better healthcare for women is good business” [10]. While not focused on endometriosis, the report highlights the massive potential healthcare industry players have to commercialize and profit from this industry. This growing commercial interest is troubling with corporations commodifying women’s health and experiences under the banner of empowerment. Feminist or social responsibility narratives–often grounded in reputational motives–corrupt health and sex/gender equality into commodities, obscuring structural inequities and exploiting vulnerable women. This situation is aggravated by the lack of regulation and access, as well as gaps in health literacy. On the contrary, the growing commercial interest could prompt progress by accelerating research, improving diagnostics and treatments, and elevating conditions such as endometriosis within public and political discourse. The challenge is therefore not to reject commercial momentum, but to strategically utilize it towards health equity, ensuring that women are recognized as partners, not products.

Amplifying female voices is imperative in this endeavor, which will also require education on endometriosis and menstrual health more broadly to start as early as is appropriate. Early education, in particular for young females, is crucial for allowing informed choices, as some of these are life-changing decisions with downstream consequences. Efforts across the globe trying to change how endometriosis is perceived and talked about are on the rise. In a randomized controlled trial in Canada, researchers were able to show that teaching adolescents about menstrual health and endometriosis improved their knowledge, understanding of endometriosis and related menstrual health issues [11]. In New Zealand, the introduction of a menstrual health and endometriosis education program in secondary schools not only raised awareness, but also prompted young women to seek specialist care earlier [12]. In the US, the ENPOWR program has expanded its endometriosis education program to school nurses, recognizing that student education alone is insufficient if the adults they turn to are not equipped to listen, recognize symptoms and act [13]. In Wales, where the menstrual cycle is already a mandatory part of the curriculum, new teaching material will be designed to include conditions such as endometriosis, signaling that menstrual health is a public health issue, not a private burden [14]. However, education cannot rest on the shoulders of girls and women alone. Boys and men too must learn alongside them so that menstrual health becomes a shared responsibility rather than a burden placed on one gender only.

Endometriosis should be a central pillar in rebuilding women’s health research agendas. Recognizing and validating patients’ experiences should be weaved into policies. Journals and publishers should support endometriosis awareness by bringing the topic to the forefront, highlighting research, and amplifying patient voices. Research efforts should focus on the development of early, noninvasive, and affordable diagnostics, the establishment of well-characterized longitudinal cohorts, and the conduct of rigorous, adequately powered clinical trials to generate meaningful therapeutic advances. Equally important, training programs that integrate hormone-based and pain‑competent care, should be made widely available and expanded. Together, these efforts will help to close longstanding gaps in endometriosis recognition, diagnosis, and management.
